# Malakoplakia presenting as Pleuropulmonary masses: A rare clinical, radiological and histopathological diagnosis

**DOI:** 10.1016/j.radcr.2021.06.070

**Published:** 2021-09-08

**Authors:** Pooja Narwani, Ishwariya Rajendran, Alex Lewington, Ayaa Eltayeb, Abdul Ganjifrockwala, Rajesh Annamalaisamy

**Affiliations:** a Department of Radiology, Royal Oldham Hospital, Pennine Acute hospitals NHS Trust, United Kingdom; b Department of Pathology, Royal Oldham Hospital, Pennine Acute hospitals NHS Trust, United Kingdom; c Royal Oldham Hospital, Pennine Acute hospitals NHS Trust, United Kingdom

**Keywords:** Pleuropulmonary Malakoplakia, Computed Tomography CT, Positron Emission Tomography PET, Pulmonary, Chest, malignancy mimic

## Abstract

Pleuropulmonary malakoplakia is a rare granulomatous inflammatory condition characterized by the accumulation of histiocytes that contain basophilic inclusions called Michaelis-Gutmann bodies . It is usually reported in patients with acquired immunodeficiency syndrome. We present clinical, radiological, pathological features and management of a rare case of pulmonary malakoplakia in an immunocompetent male patient with a past history of empyema treated with surgical decortication. Clinically, the patient presented with shortness of breath, productive cough and lethargy. On imaging, Computed Tomography of Thorax showed multiple nodular lung masses and nodular pleural thickening with marked Fluorodeoxyglucose Positron Emission Tomography avidity raising suspicion of advanced pulmonary malignancy. Characteristic Michaelis-Gutmann bodies were identified on histopathology, confirming the diagnosis of malakoplakia. The patient was medically managed with a long course of antibiotics. On follow-up, there was a significant clinical and radiological improvement. Pulmonary malakoplakia is a rare entity, with very few cases reported worldwide, and even fewer in immunocompetent individuals.

## Case report

**Fig. 1:**
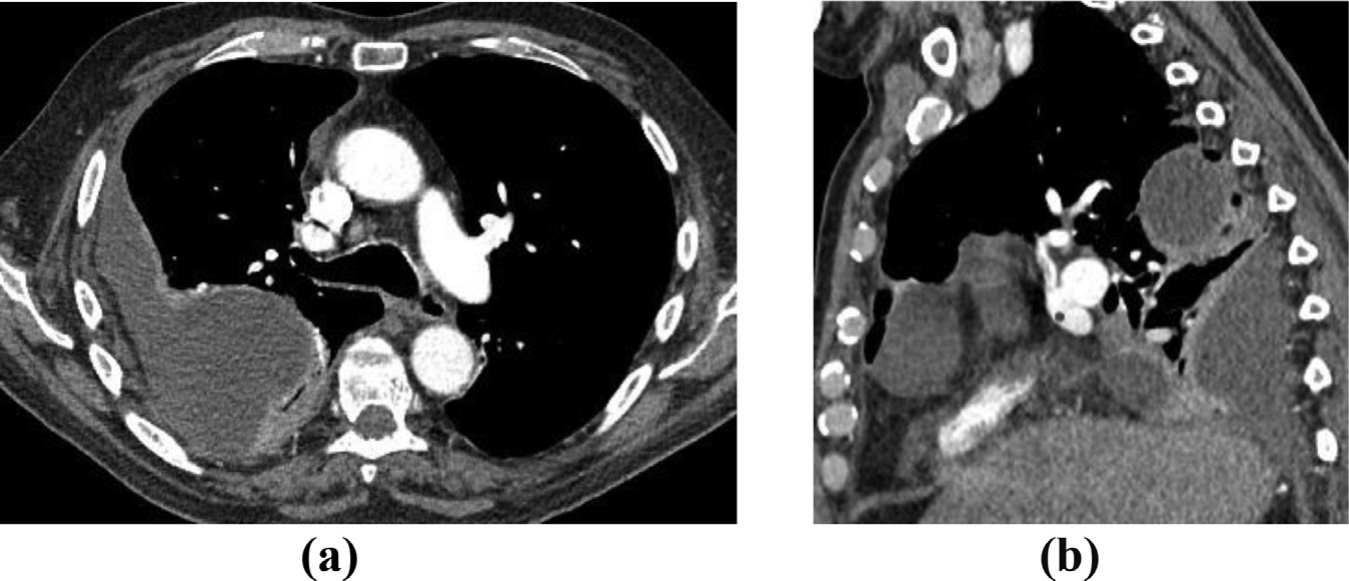
CT Thorax images in axial (a) and sagittal (b) planes showing a loculated right pleural effusion with enhancement of both layers of pleura suggesting an empyema.

A 72-year old male was referred to the pleural clinic for right-sided empyema diagnosed on CT Thorax (Fig. 1a and b). The patient had a 20-pack year history of smoking and past medical history of hypertension. Aspirated pleural fluid was exudative containing inflammatory cells and no acid-fast bacilli (AFB). The cultures grew Escherichia coli. Clinical improvement was noted after a course of antibiotics.

**Fig. 2:**
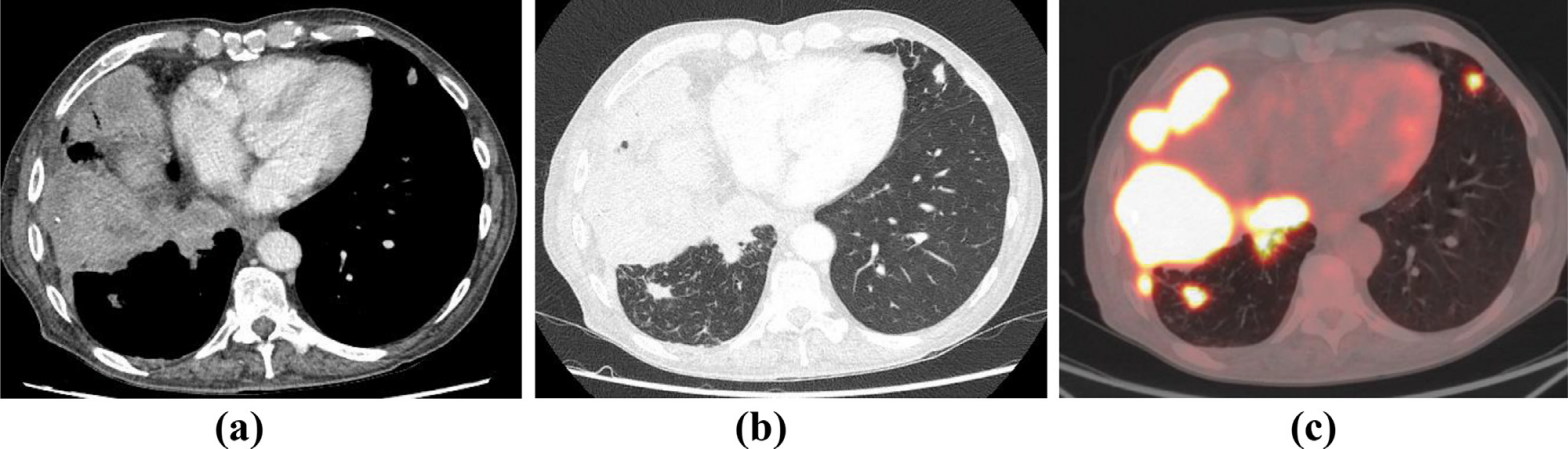
Axial CT images of thorax in (a) mediastinal and (b) lung windows show nodular thickening of right pleura, predominantly over the right hemidiaphragm suggestive of metastatic lung malignancy. Also note volume reduction of right hemithorax due to previous empyema. (c) PET CT axial fused image reveals metabolically active pleural disease. Note FDG avid lung nodule in the lingula. PET complemented CT diagnosis of an aggressive primary pulmonary malignancy.

**Fig. 3:**
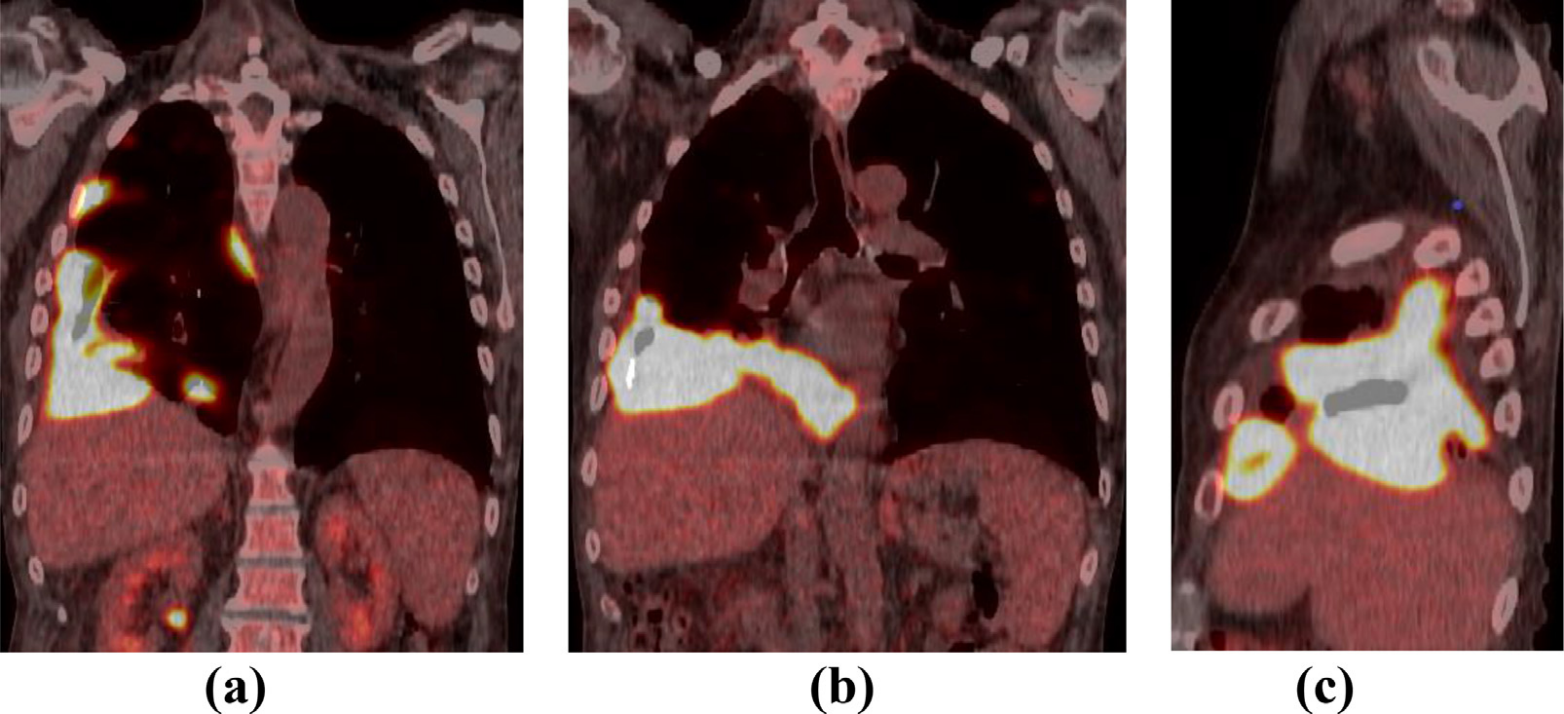
PET CT images in coronal and sagittal planes showing extensive involvement of the right lung and pleura.

Few months later, the patient presented with breathlessness, lethargy, productive mucoid cough with small volume haemoptysis and weight loss over 6 kg. The patient was upgraded to an urgent cancer pathway and further investigations such as chest x-ray, CT scan of thorax (Fig. 2a and b) and Positron Emission Tomography (PET) CT (Figs. 2c and 3) were performed.

**Fig. 4:**
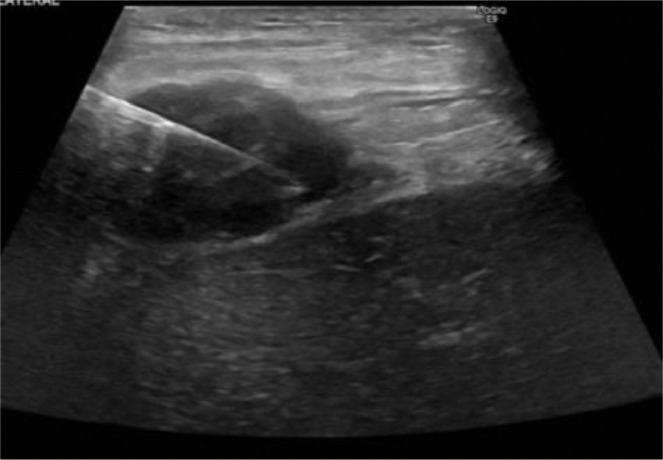
Ultrasound guided biopsy of right sided pleural thickening with core biopsy needle.

An ultrasound-guided biopsy of right-sided pleural thickening was performed (Fig. 4).

**Fig. 5:**
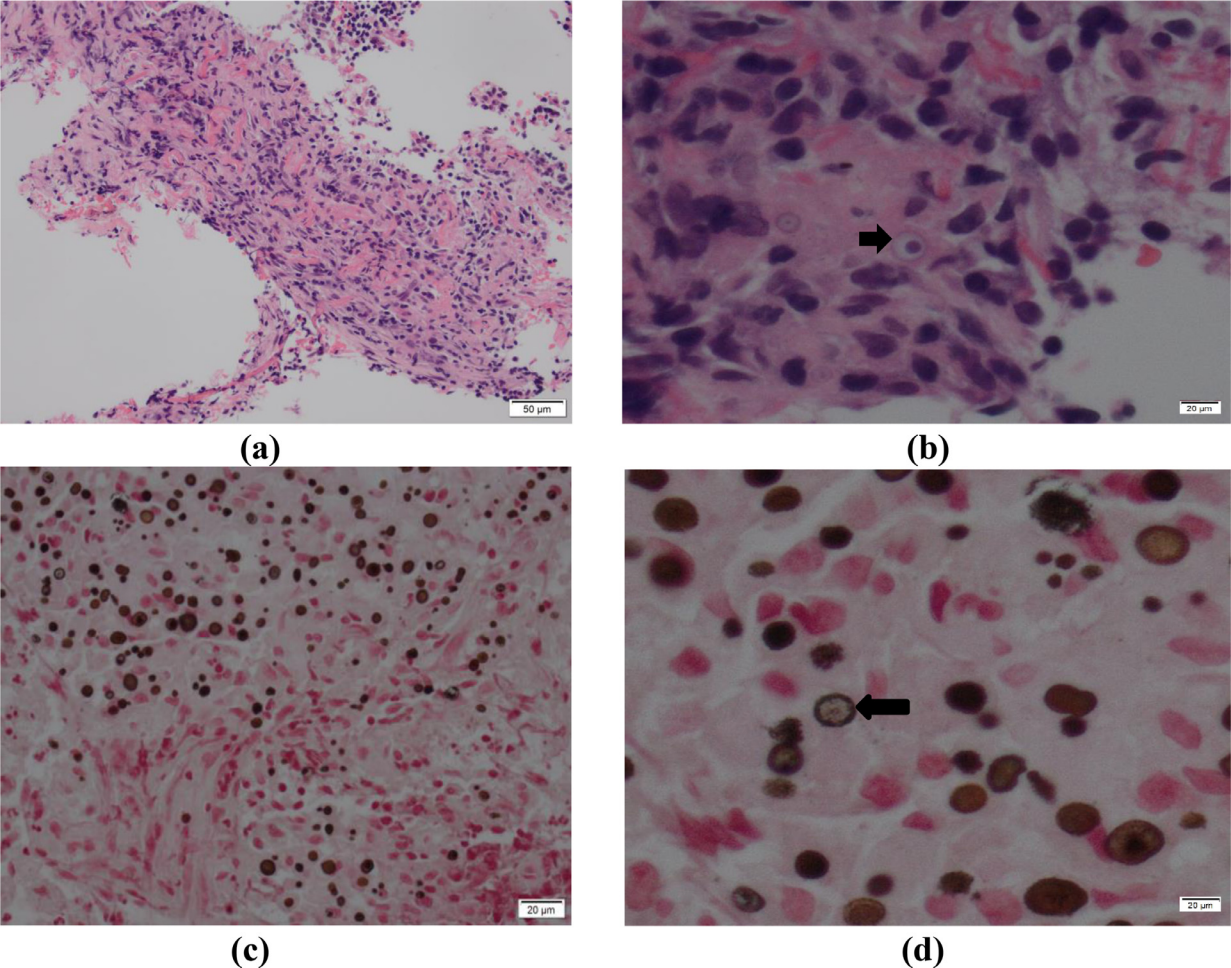
**(a):** Dense lymphoplasmacytic infiltrate of lung tissue with scattered MGB, H&E stain x100 magnification, **(b)** Arrow: MGB within granular histocytes typical of Von Hansemann's histocytes, H&E stain x400 magnification **(c)** Positive Von Kossa stain staining MGB, x200 magnification **(d)** Arrow: MGB stained with Von Kossa showing intracellular round laminations with a targetoid appearance, x400 magnification.

The core biopsies were largely replaced by fibrosis. Within the fibrosis, dense lymphoplasmacytic infiltrate and Von Hansemann histiocytes containing granular eosinophilic cytoplasm were seen. Von Hansemann histocytes are known to destroy alveoli leaving fibrotic interstitium behind. Many of these granular histocytes contained concentrically layered targetoid basophilic inclusions typical of Michaelis-Gutmann calcific bodies (Fig. 5).

The histochemical profile demonstrated strong and diffuse positivity for periodic acid-Schiff stain (PAS) granular cytoplasmic staining, confirming the presence of PAS+ bacteria within the von Hansemann histocytes. The MGB were positive for Von Kossa stain, a staining method used to highlight calcium deposits (Image 7c and 7d). Grocott stain, a staining method for highlighting the carbohydrate wall of fungi, was negative for fungal elements, and no granulomas were identified. Differential diagnoses based on morphological features using hematoxylin and eosin (H&E) included:

• nonspecific inflammation
• pulmonary malakoplakia
• granular cell tumor
• Whipple's disease
• atypical mycobacterial infection

Pulmonary malakoplakia was diagnosed based on the presence of MGBs confirmed with Von Kossa stain.

**Fig. 6:**
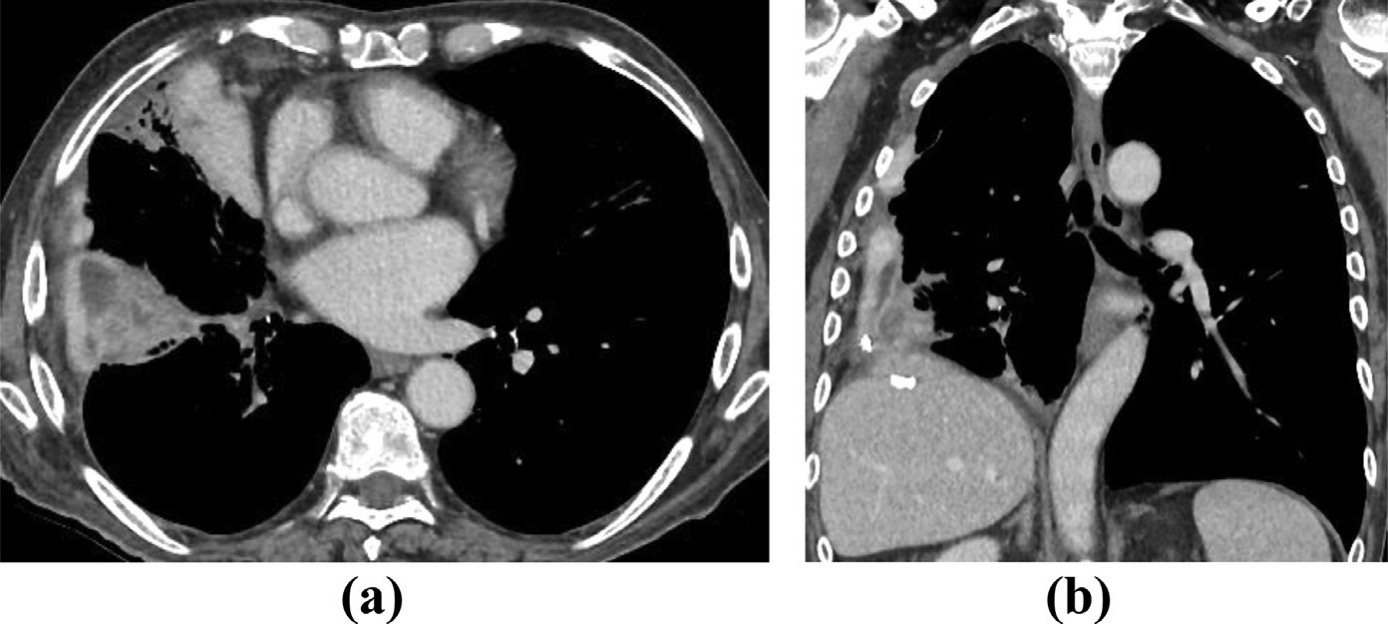
CT scan thorax (axial and coronal) shows significant improvement of pleural and parenchymal nodular masses with residual pleural thickening containing a focus of calcification.

A decision was made to step down from the cancer pathway and continue treatment with a long 10-week course of ciprofloxacin and carbocisteine. The patient improved clinically, and interim CT showed regression of soft tissue masses and nodularity in the right hemi thorax on follow-up (Fig. 6).

Performing image-guided biopsy completely changed the diagnosis from advanced lung malignancy on imaging to a potentially curable inflammatory condition.

## Discussion

Pleuropulmonary malakoplakia is a rare granulomatous inflammatory condition characterized by the accumulation of granulomatous masses mimicking malignancy. It is essentially a histopathological diagnosis characterized by the accumulation of macrophages containing characteristic intracellular or extracellular inclusions referred to as MGB. The term malakoplakia is derived from Greek word ‘malakos’ meaning soft, and the word ‘plakos’ meaning plaque. The condition was initially described by Michaelis and Gutmann in 1902 [1] but first reported by Professor Von Hansemann in 1901 [2].

Pathogenesis is uncertain but believed to be due to defective lysosomal activity leading to defective phagocytosis and accumulation of intra-cytoplasmic deposition of fragments from bacteria, iron, and calcium, which would form the MGB [3]. It is postulated that inadequate phagocytosis occurs due to low levels of cyclic guanine monoamine phosphatase.

Genitourinary tract is the most common site representing 58%-80% of cases, with most cases reported in females and associated with infection by gram-negative bacteria such as Escherichia coli and Klebsiella sp. Gastrointestinal tract and retroperitoneal sites are the second most common sites, contributing to 12%-15% of cases. Other rare sites include lung, liver, bone, skin and uterus. Other organisms associated with malakoplakia are:

• Rhodococci equi
• Proteus mirabilis
• Pseudomonas aeruginosa
• Yersinia
• Staphylococcus aureus
• Fungal infections

Most patients have some form of immunosuppression, including solid organ transplant, autoimmune disease requiring steroid use, or chemotherapy. Alcoholism, chronic systemic disease, poorly controlled diabetes and malignancy can also play a role.

Pulmonary malakoplakia is a rare diagnosis with very few cases reported worldwide, the first case reported by Gupta et al. in 1972 [4]. It is most commonly associated with Rhodococci equi in immunocompromised patients; however, rare cases with other organisms have been reported [5,6]. So far, only 38 cases of pulmonary malakoplakia have been noted in English literature [7]. The average age of diagnosis is 50 years with no sex preponderance. Immunosuppression due to HIV and other underlying diseases play a crucial role in pathogenesis. In our case, Escherichia coli organism was isolated from pleural fluid, with the patient having a past history of treated pleural empyema.

Clinically and radiologically, pulmonary malakoplakia can mimic tumors, abscesses or infections such as tuberculosis [8,9]. In our case, malakoplakia mimicked an aggressive metastatic pulmonary malignancy.

Histologically, there is a collection of foamy histiocytes or von Hansemann cells containing basophilic inclusion with clear halos called MGB. These inclusions are phagolysosomes containing undigested bacterial fragments. Biopsy is diagnostic.

The treatment is mostly conservative and consists of a course of antibiotic depending on the organism isolated in culture.

## Teaching points

Malakoplakia most commonly affects the genitourinary system in female patients with chronic bladder infection.

Clinical, radiological and histopathological correlation is essential for diagnosing pulmonary malakoplakia to prevent unnecessary surgical intervention. Treatment of malakoplakia is usually conservative by a long course of antibiotics.

Although rare, pulmonary malakoplakia should be considered in unusual infections occurring at unusual sites, especially in immunocompromised hosts.

## Consent for publication

Written informed consent taken for publication of case report.

## Patient consent

A written informed consent for the publication of this case has been taken from the patient. A consent form can be attached if required.
